# LincK contributes to breast tumorigenesis by promoting proliferation and epithelial-to-mesenchymal transition

**DOI:** 10.1186/s13045-019-0707-8

**Published:** 2019-02-22

**Authors:** Jing Li, Yajing Hao, Wenzhe Mao, Xiaowei Xue, Pengchao Xu, Lihui Liu, Jiao Yuan, Dongdong Zhang, Na Li, Hua Chen, Lin Zhao, Zhao Sun, Jianjun Luo, Runsheng Chen, Robert Chunhua Zhao

**Affiliations:** 1 Institute of Basic Medical Sciences Chinese Academy of Medical Sciences, School of Basic Medicine Peking Union Medical College, Peking Union Medical College Hospital, Center of Excellence in Tissue Engineering Chinese Academy of Medical Sciences, Beijing Key Laboratory (No. BZO381), Beijing, 100005 China; 2 Key Laboratory of RNA Biology, Institute of Biophysics, Chinese Academy of Sciences, Beijing, 100101 China; 3 Department of Pathology, Peking Union Medical College Hospital, Chinese Academy of Medical Sciences and Peking Union Medical College, Beijing, 100005 China; 4 Department of Oncology, Peking Union Medical College Hospital, Chinese Academy of Medical Sciences and Peking Union Medical College, Beijing, 100005 China

**Keywords:** lncRNA, Breast cancer, miR-200, Tumor microenvironments, Epithelial-to-mesenchymal transition

## Abstract

**Background:**

Increasing evidence has demonstrated that mesenchymal stem cells (MSCs) play a role in the construction of tumor microenvironments. Co-culture between tumor cells and MSCs provides an easy and useful platform for mimicking tumor microenvironments and identifying the important members involved in tumor progress. The long non-coding RNAs (lncRNAs) have been shown to regulate different tumorigenic processes. In this study, we aimed to examine functional lncRNA deregulations associated with breast cancer malignancy instigated by MSC-MCF-7 co-culture.

**Methods:**

The microarrays were used to profile the expression changes of lncRNAs in MCF-7 cells during epithelial-mesenchymal transition (EMT) induced by co-culture with MSCs. We found that an intergenic lncRNA KB-1732A1.1 (termed LincK, partly overlapped with GASL1) was significantly elevated. To investigate the biological function of LincK, the expression of EMT markers, cell migration, invasion, proliferation, and colony formation were evaluated in vitro and xenograft assay in nude mice were performed in vivo. Furthermore, we detected LincK expression in clinical samples using RNAscope® technology and verified aberrant expression of LincK in breast cancer data sets from The Cancer Genome Atlas (TCGA) by bioinformatic analysis. The underlying mechanisms of LincK were investigated using mRNA microarray analyses, Western blot, RNA pull down, and RNA immunoprecipitation.

**Results:**

LincK induced an EMT progress in breast cancer cells (BCC) MCF-7, MDA-MB-453, and MDA-MB-231. The depletion of LincK decreased the growth, migration, and invasion in BCC, whereas the overexpression of LincK exerted the opposite effects. Moreover, knockdown of LincK repressed tumorigenesis, and ectopic expression of LincK promoted tumor growth in MCF-7 xenograft model. LincK ablation in MDA-MB-231 cells dramatically impaired lung metastasis when incubated intravenously into nude mice. Further, LincK was frequently elevated in breast cancer compared with normal breast tissue in clinical samples. Mechanistically, LincK may share common miRNA response elements with PBK and ZEB1 and regulate the effects of miR-200 s.

**Conclusion:**

LincK plays a significant role in regulating EMT and tumor growth and could be a potential therapeutic target in breast cancer.

**Electronic supplementary material:**

The online version of this article (10.1186/s13045-019-0707-8) contains supplementary material, which is available to authorized users.

## Introduction

Tumor progression is a highly complex process that involves both intrinsic genetic changes and extrinsic signals mediated via specialized tumor microenvironments [1]. Tumor cells can actively recruit a variety of cell types, including mesenchymal stem cells (MSCs), into the tumor microenvironment [2–4]. MSCs are pluripotent progenitor cells that contribute to the maintenance and regeneration of a variety of connective tissues, including bone, adipose, cartilage, and muscle [5, 6]. Accumulating evidence suggests that the cross-talk between MSCs and breast cancer cells may impact tumorigenesis, growth, and metastasis [7–9]. Previously, we co-cultured adipose-derived (AD)-MSCs with the breast cancer cells MCF-7 in vitro and found that the co-cultured MCF-7 cells displayed increased migratory and invasive capacities and underwent epithelial-mesenchymal transition (EMT) changes in a time-dependent manner [10]. Further investigation of the molecular mechanism underlying the MSC-tumor interplay may facilitate the development of new interventions targeting tumor growth and metastasis.

Long noncoding RNAs (lncRNAs) are defined as RNA transcripts longer than 200 nucleotides that lack apparent protein coding potential. Genome-wide analyses based on the basis of GENCODE annotations have identified approximately 14,000 lncRNA genes in the human genome [11]. Numerous studies have revealed that lncRNAs play important roles in multiple biological processes, including development, differentiation, and carcinogenesis. For example, HOTAIR induces breast cancer metastasis by operating as a tether that links EZH2 (PRC2) and LSD1, thereby coordinating their epigenetic regulatory functions [12, 13]. lncRNA-ATB mediates TGF-β-induced EMT and colonization by competitively binding the miR-200 family and IL-11 mRNA, thus promoting the invasion-metastasis cascade in hepatocellular carcinoma [14]. MALAT1 is a conserved non-coding transcript whose expression has been associated with metastasis and reduced survival in patients with multiple tumor types [15]. More importantly, MALAT1 is not required for normal development, which makes it a potential therapeutic target against cancer progression [16]. To date, only a few lncRNAs have been well studied for the cause and progression of cancers.

In this study, we aimed to identify the potential lncRNA deregulations associated with breast cancer malignancy instigated by MSC-MCF-7 co-culture. We profiled the expression changes of lncRNAs in MCF-7 cells during EMT induced by co-culture with hAD-MSCs and observed an intergenic lncRNA KB-1732A1.1 (transcript uc003ykw.2, termed LincK) that was significantly elevated. Linck partly overlapped with lncRNA GASL1, which was found to inhibit cell cycle in some cancer cells [17]. Here, we reported for the first time the role of LincK in controlling EMT and proliferation in breast cancer.

## Materials and methods

### Cell lines

The cell lines MCF-7, 293 T, MDA-MB-453, and MDA-MB-231 were directly obtained from the Cell Resource Center, Peking Union Medical College (which is the headquarters of the National Infrastructure of Cell Line Resource, NSTI).

### Isolation and expansion of adipose-derived hAD-MSCs

Human adipose tissue was obtained from patients undergoing tumescent liposuction according to the procedures approved by the Ethics Committee at the Chinese Academy of Medical Sciences and Peking Union Medical College. Isolation of hAD-MSCs was followed by procedures as previously reported [5].

### Cell-cell co-culture and enzyme-linked immunosorbent assay

A Transwell® chamber with a 0.4-μm pore size permeable membrane (Corning) was used to co-culture the MCF-7 cells, MDA-MB-453, or MDA-MB-231 with the hAD-MSCs at a ratio of 1:1. The cells were continuously passaged when needed.

To detect the secretion of interleukin-6 (IL-6), TGF-β, and VEGF, hAD-MSCs with or without co-culture with MCF-7 for 7 days were plated in 6-well plates at a cell density of 2 × 10^5^/ml. After incubation in serum-free medium for 24 h, the supernatant was collected for analysis using enzyme-linked immunosorbent assay (ELISA) kits (NeoBioscience, China) according to the manufacturer’s instructions.

### Microarray and data analysis

Total RNA was isolated using Trizol from MCF-7 cells cultured alone (0 day) and co-cultured with hAD-MSCs for the indicated times (2, 4, 6, 8, and 10 days). Sample processing and hybridization were conducted as described in a previous report [18, 19].

For the siRNA-mediated knockdown of LincK in MCF-7 cells, RNA was extracted 48 h after si-NC (Negative Control), si-LincK1, and si-LincK2 transfection. Three biological replicates were done. A fold change ≥ 2 in its expression level and a *p* value ≤ 0.05 were chosen as the cut-off criteria. All microarray data were uploaded to Gene Expression Omnibus (accession number, GSE109007 and GSE109008).

### siRNA, plasmids, and virus infection

siRNAs used to knockdown target lncRNAs or mRNAs were designed by the online tool (BLOCK-iT™ RNAi Designer) and synthesized by GenePharma (Suzhou, China). For overexpression, the full length of LincK was inserted into lentivirus expression vector PCDH-CMV-MCS-EF1-puro (termed as LV-Control thereafter). For knockdown, lentivirus shRNA expression vectors targeting the same sequences as siRNAs were constructed and packaged by GenePharma.

### In vitro migration and invasion assay

For MCF-7 and MDA-MB-453 (with LincK overexpression, knockdown, or their corresponding controls), tumor cells were co-cultured with hAD-MSCs for 2 weeks before being subjected to the migration and invasion assay. Then, tumor cells were resuspended in 200-μl serum-free medium at a density of 1 × 10^6^/ml and seeded into the upper chamber of 24-well Transwell chambers (8-μm pore, costar) coated without (migration) and with (invasion) Matrigel (BD Biosciences). The lower chambers were filled with 600 μl of medium containing 20% FBS. After 24 h (migration) or 36 h (invasion), cells on the lower surface of the inserts were stained with 0.1% Crystal Violet.

For MDA-MB-231 cells, 5 × 10^4^ cells were added into the top chamber and permitted to migrate for 8 h; 2 × 10^5^ cells were seeded into the top chamber coated with Matrigel and permitted to invade for 24 h. Three randomly selected fields per filter were counted.

### Colony formation assay

For MCF-7 and MDA-MB-231 cell lines, 2000 cells were suspended in 5 ml complete medium and seeded in a 60-mm dish. For MDA-MB-453 cell line, 2 × 10^4^ cells were cultured in a 6-mm dish. After 2 weeks, colonies were stained by 0.1% crystal violet. Photos of colonies were taken by Cannon EOS 600D and number of colonies were analyzed by ImageJ software.

### RNA extraction and quantitative reverse transcription-polymerase chain reaction

Total RNA was extracted using the Trizol reagent (Invitrogen), and quantitative reverse transcription-polymerase chain reaction (qRT-PCR) analysis of mRNA and miRNAs was performed as we previously described [5]. All the primer sequences are listed in Additional file 1: Table S1.

### Western blot

Western blotting was performed as we previously described [5]. Antibodies against the following proteins were obtained as indicated: ZEB1, E-cadherin, N-cadherin, ZO-1, Vimentin, and GAPDH (Proteintech, China); PBK, p38 MAPK, and phosphorylated p38 MAPK (Cell Signaling Technology®).

### Cell proliferation assay

Cells were plated in 96-well plates (2000 cells/well). Cell proliferation was determined every 24 h for 5 days according to the manufacturer’s instructions. Briefly, 10 μl of MTS (#G3582, Promega) was added to each well. After incubation at 37 °C for 1 h, the absorbance at 490 nm was detected.

### BrdU proliferation assay

Cell proliferation was monitored using the BrdU-ELISA kit (#11647229001, Roche) according to the manufacturer’s instructions. Briefly, 1 × 10^4^ cells were plated in 96-well plates for 48 h and then labeled with BrdU for 2 h. After incubation with BrdU antibody-peroxidase (POD), photometric detection was performed at 370 nm wavelength.

### Northern blot

Northern blots were performed using the DIG Northern Starter Kit (#12039672910, Roche) as we described previously [20]. Digoxigenin (DIG)-labeled LNA probes were designed using online software (Stellaris probe designer) and synthesized by Exiqon.

### 5′ and 3′ rapid amplification of cDNA ends

The transcriptional initiation and termination sites of LincK were detected using the FirstChoice RLM-RACE Kit (#AM1700, Ambion) according to the manufacturer’s instructions. The primer sequences are listed in Additional file 1: Table S1.

### Subcellular fractionation

The separation of the nuclear and cytosolic fractions was performed using the NE-PER Nuclear and Cytoplasmic Extraction Reagents (#78833, Thermo Scientific) according to the manufacturer’s instructions. RNA was extracted, and qRT-PCR was performed to assess the relative proportion in the nuclear and cytoplasmic fractions.

### Dual luciferase reporter assay

Wild types of full-length LincK and 3′UTR of ZEB1 and PBK were obtained by PCR or RT-PCR and cloned into the luciferase reporter vector psiCHECK2 (Promega). Mutants were prepared by deleting of 16 base-pair binding sequences of miR-200b. The cells were harvested 24 h after transfection, and Renilla and firefly luciferase activity were analyzed using the Dual-Luciferase® Reporter Assay System (#E1910, Promega).

### RNA immunoprecipitation assay

The Ago2-LincK RNA immunoprecipitation (RIP) assay was performed with the EZ-Magna RIP Kit (Millipore). The AGO2 antibody was purchased from Millipore (#03-110, Merck). The Flag-MS2bp-MS2bs-based RIP assay was conducted according to previous reports [21]. Briefly, 293 T cells were co-transfected with pcDNA3-Flag-MS2bp and LincK-MS2bs or mutant-MS2bs, and the cell lysates were harvested with IP lysis buffer (#87787, Pierce) after 48 h. Anti-FLAG® M2 Magnetic Beads (Sigma) was used to immunoprecipitate Flag-MS2bp fusion protein. The co-precipitated RNAs associated with Flag-MS2bp were extracted by Trizol reagent (Invitrogen) and detected by qRT-PCR.

### RNA pull down

Biotin-labeled RNAs were transcribed in vitro with the Biotin RNA Labeling Mix (Roche) and T7 RNA Pol II (Promega). Whole-cell extract, prepared from 1 × 10^7^ cells in 1 ml of RIP buffer (150 mM KCl, 25 mM Tris (pH 7.4), 5 mM EDTA, 0.5 mM DTT, and 0.5% NP-40) containing RNase and protease inhibitors, was mixed with 3 μg of biotinylated RNA and incubated at RT for 1.5 h, then followed by the addition of 50 μl washed Streptavidin Dynabeads to incubate for another 1.5 h. The beads were washed for 5 × 5 min with RIP buffer containing 0.5% sodium deoxycholate. RNA associated with the beads were extracted by Trizol reagent (Invitrogen) and detected by qRT-PCR.

### Clinical specimens, tissue microarrays, and RNAscope®

Breast tissue specimens of normal, benign, and invasive breast cancers were obtained with informed consent from patients in Peking Union Medical College Hospital (Beijing, China) during Oct 2016 to Feb 2017. All specimens were collected using the protocols approved by the Ethics Committee of Peking Union Medical College Hospital. TMA were prepared by Zhongshan Golden Bridge Biotechnology CO., LTD (Beijing, China). The RNAscope® probe targeting LincK was designed and synthesized by Advanced Cell Diagnostics, and the expression of LincK was detected using the RNAscope 2.0 HD detection kit (Brown) according to the manufacturer’s instructions [22].

### Xenograft assay in nude mice

For the subcutaneous model, MCF-7 cells with LincK knockdown, LincK overexpression, and the corresponding controls (stably transduced with lentivirus vectors) were mixed with hAD-MSCs at the ratio of 5:1. After washing for three times with PBS, these cells were resuspended in PBS supplemented with 10% Matrigel at a concentration of 6 × 10^6^/100 μL. Female Balb/c nude mice (5–6 weeks old) were then injected with 100 μl of cell suspension subcutaneously. To promote the tumorigenesis, the mice were injected intraperitoneally with 1 mM estradiol cypionate (MCE, # HY-B1100, 100 μl/mouse) on the day of tumor incubation. The tumor sizes were measured every week for 5 weeks, and the volumes (in cubic millimeters) were calculated according to the equation: width^2^ × length × 0.5.

For metastasis assay (colonization), 1 × 10^6^ MDA-MB-231 cells (shCtrl or shLincK2 in 100 μl of PBS suspension) were injected intravenously into immunodeficiency mice (*n* = 6). Lungs were subsequently collected at week 7, fixed in 10% formalin, and embedded in paraffin. Serial pathological sections from the embedded tissues were stained by standard HE procedure to detect metastasis.

## Results

### LincK was upregulated during co-culture-induced EMT

We previously reported that transwell co-culture with hAD-MSC cells can induce an EMT process in MCF-7 cells [10]. To identify EMT-relevant lncRNAs, we conducted transcriptome microarray analyses of the MCF-7 cells at days 0, 2, 4, 6, 8, and 10 after co-culture. Functional enrichment analysis of the differentially expressed coding genes showed that they were significantly enriched in the TGF-β-signaling associated pathway, including in cell adhesion, SMAD protein phosphorylation, and cell migration (Additional file 2: Figure S1A). Moreover, the expression levels of epithelial markers were downregulated, and those of mesenchymal markers were upregulated in the MCF-7 cells after co-culture with MSCs (Additional file 2: Figure S1B). Additionally, gene set enrichment analysis (GSEA) revealed that differential expressed genes were significantly correlated with TGF-β response gene sets defined by four independent studies [23–25]. These results were consistent with our previous study and confirmed that co-culture clearly induced EMT in MCF-7 cells.

**Fig. 1:**
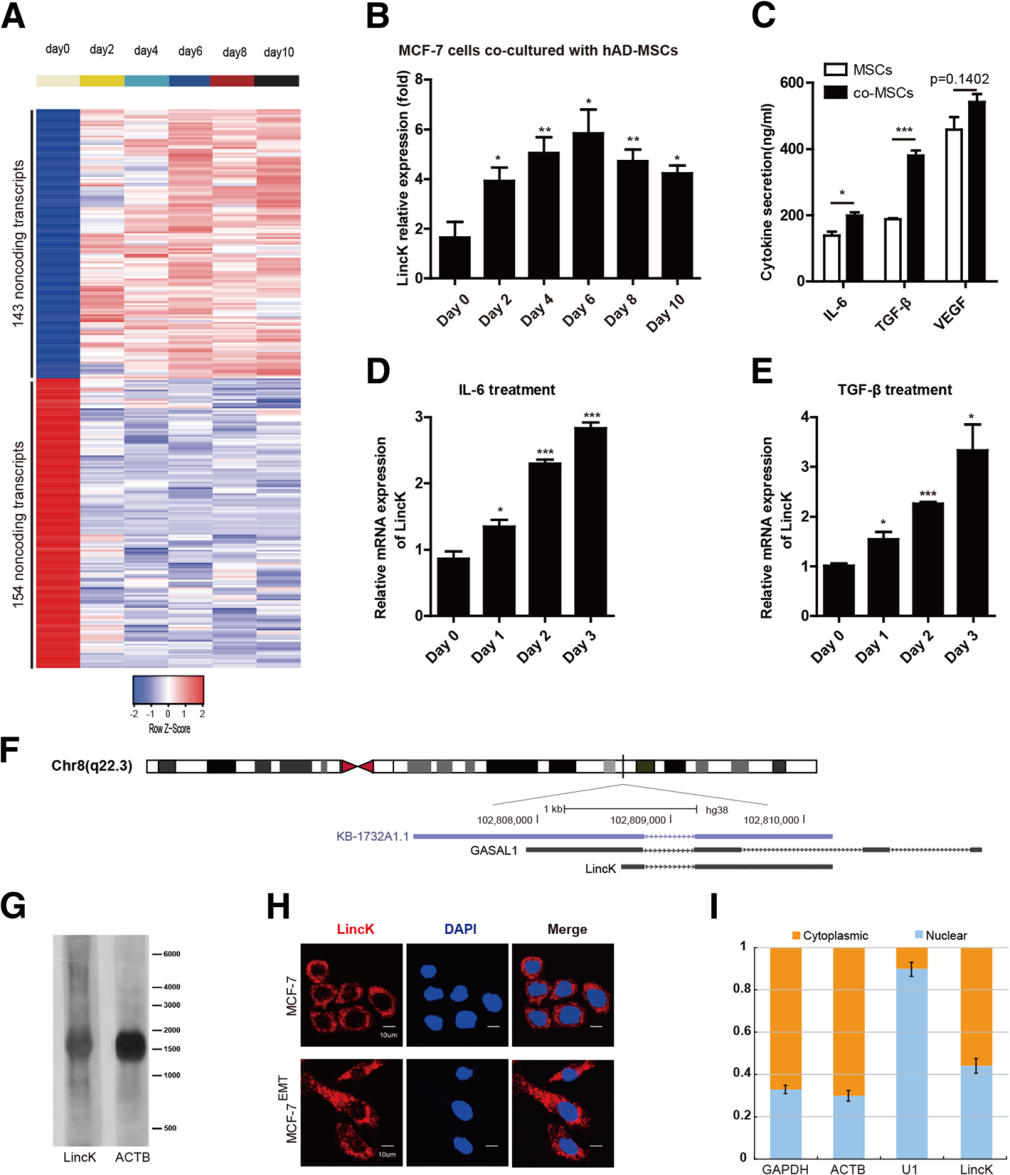
LincK was upregulated during co-culture induced EMT. **a** Heat map of differential expressed lncRNA from microarray data. 143 upregulated and 154 downregulated lncRNAs were represented in MCF-7 cells co-cultured with hAD-MSCs (for indicated days) compared with MCF-7 cells cultured alone (day 0). **b** Relative expression of LincK were detected by real-time RT-PCR in MCF-7 cells co-cultured with hAD-MSCs and compared with MCF-7 cells cultured alone (day 0). Relative gene expressions were normalized to GAPDH unless noted otherwise in this study. Results were shown as means ± S.D. of triplicate experiments. **c** Cytokine secretion of hAD-MSCs before and after co-culture with MCF-7 was detected by ELISA. Results were shown as means ± S.D. **d**, **e** Relative expression of LincK in MCF-7 cells after treated with IL6 (50 ng/ml, **d**) or TGF-β1 (10 ng/ml, **e**) were detected by qRT-PCR. Data were shown as mean ± S.D. **f** Schematic annotation of LincK genomic locus on chromosome 8. KB1732A1.1 (uc003ykw.2, top), GASL1 (middle), LincK (bottom) were shown. Rectangles represented exons. **g** Northern blot of LincK and ACTB transcripts in MCF-7 cells. **h** Representative images of subcellular localization of LincK detected by RNA-FISH assays in MCF-7 and MCF-7^EMT^ (co-culture induced EMT) cells. DAPI, 4′, 6-diamidino-2-phenylindole. Scale bar, 10 μm. **i** Fractionation of MCF-7 cells followed by qRT-PCR. GAPDH and ACTB served as cytoplasmic mRNA controls. U1 served as a nuclear RNA control. Bars indicate S.D., *n* = 3. *p < 0.05; **p < 0.01; ***p < 0.001 (Student’s *t* test)

Next, we analyzed lncRNA expression profiles and found 143 upregulated and 154 downregulated lncRNAs (Fig. 1a). The distribution of these lncRNAs was displayed in Additional file 2: Figure S1D. Among these differentially expressed lncRNAs, we observed that one transcript, KB-1732A1.1 (uc003ykw.2), showed a robust elevated expression in the MCF-7 cells upon co-culture. The upregulation of LincK during co-culture-induced EMT was further validated by qRT-PCR (Fig. 1b). MSCs can secrete multiple cytokines including TGF-β and interleukin 6 (IL-6), which can be further enhanced by co-culturing with MCF-7 (Fig. 1c). TGF-β and IL-6 have been reported to induce EMT in several tumor cell types [26, 27]. Additionally, treatment with TGF-β1 and IL-6 also can increase the expression of LincK in MCF-7 cells (Fig. 1d, e). Because LincK responded to multiple EMT inducers, we speculated that it might be involved in EMT and have a biological function in breast cancer.

### Identification and characterization of LincK

LincK was previously annotated as a long non-coding RNA in GENCODE and mapped to the long arm of chromosome 8q22.3. We then examined the coding potential of LincK using in silico prediction, ribosome profiling data, and in SmProt database (Additional file 3: Figure S2A and S2B) [28]. These data consistently showed that LincK had no ability to code a protein. To determine both ends of this transcript, we performed 5′ and 3′ rapid amplification of cDNA ends (RACE) assays in the MCF-7 cells. Consistent with the annotation of KB1732A1.1 (uc003ykw.2), LincK was polyadenylated and contained two exons. Contrasting with the annotation, the first exon was considerably shorter in length (150 vs. 1200 bp, Fig. 1f). Furthermore, the Northern blot identified LincK in the MCF-7 cells, thus confirming the RACE results (Fig. 1g). To assay the subcellular distribution of LincK, we performed RNA fluorescence in situ hybridization (RNA-FISH) and found that most of the LincK transcripts were located in the cytoplasm (Fig. 1h). Moreover, we verified this observation by quantifying the expression ratio of LincK in cytoplasmic and nuclear fractions with qRT-PCR (Fig. 1i). Taken together, LincK is a long noncoding RNA that is l.2 kb in length and mainly distributed in cytoplasm.

### LincK induced EMT in breast cancer cells

**Fig. 2:**
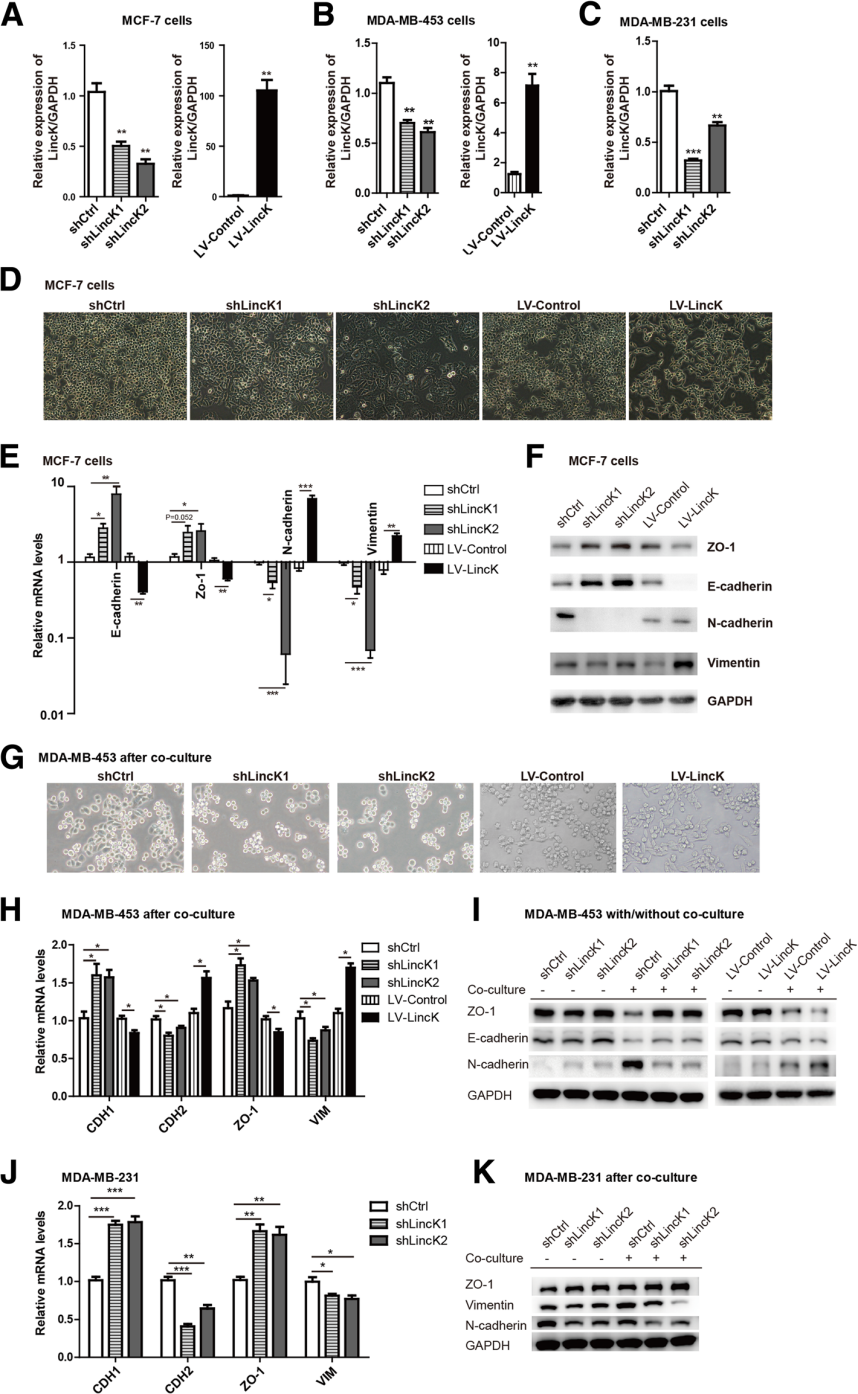
LincK promoted EMT programs of breast cancer cells in vitro. **a**–**c** Stable cell lines with LincK knockdown or ectopic expression were established using lentivirus transduction followed by puromycin selection in MCF-7 (**a**), MDA-MB-453 (**b**), and MDA-MB-231 (**c**). The expressions of LincK were detected by qRT-PCR. shCTRL, negative control; shLincK1 and shLincK2, shRNAs against LincK; LV-Control, overexpression empty vector; LV-LincK, overexpression of LincK. Data were shown as means ± S.D. **d**, **g** The morphology of MCF-7 cells (**d**) and MDA-MB-453 cells (**g**, after co-culture with hAD-MSCs for 2 weeks) with LincK knockdown and overexpression examined by phase-contrast microscopy (× 200 magnification). **e**, **h**, **j** qRT-PCR of EMT markers in MCF-7 (**e**), MDA-MB-453 (**h**), and MDA-MB-231 (**j**) with LincK knockdown or overexpression. Data were shown as means ± S.D. (*n* = 3). **f**, **i**, **k** Western Bolt assay of EMT markers in MCF-7 (**f**), MDA-MB-453 (**i**), and MDA-MB-231 (**k**) with LincK knockdown or overexpression. Error bars represent the mean ± S.D. of triplicate experiments. Statistical differences were analyzed using Student’s test (***p < 0.001; **p < 0.01; *P < 0.05)

To investigate the function of LincK in breast carcinoma, we stably knocked down or overexpressed LincK by lentivirus infection in MCF-7 cells, MDA-MB-453, and MDA-MB-231 cells. The expression of LincK was confirmed by RT-PCR (Fig. 2a–c). Then, we examined the effect of LincK on cell phenotypes. In the MCF-7 cells, overexpression of LincK induced mesenchymal-like morphology, and knockdown of LincK induced more epithelial-like features (Fig. 2d). Ectopic expression of LincK caused decreased expression of the epithelial marker CDH1/E-cadherin and ZO-1 and increased expression of the mesenchymal markers CDH2/N-cadherin and Vimentin. Conversely, knockdown of LincK resulted in increased expression of CDH1/E-cadherin and ZO-1 and decreased expression of CDH2/N-cadherin and Vimentin. The assessment of the mRNA levels showed consistent changes with the protein levels (Fig. 2e, f). Moreover, knockdown of LincK in MCF-7 cells dramatically impeded co-culture-induced EMT as evidenced by the expression of EMT markers (Additional file 4: Figure S3A and B).

The MDA-MB-453 cells appeared round in shape. Overexpression and knockdown of LincK did not cause the typical morphology changes of EMT in MDA-MB-453 (not shown). However, after co-culture with MSCs for 2 weeks, morphological changes were observed in these cells (Fig. 2g). Adhesion molecules were detected in MDA-MB-453 by both qRT-PCR and Western blot (Fig. 2h, i). Knockdown of LincK resulted in an upregulation of the epithelial marker CDH1/E-cadherin and ZO-1, and overexpression of LincK led to the upregulation of the mesenchymal marker CDH2/N-cadherin.

Then we performed loss-of-function experiment of LincK in mesenchymal-like MDA-MB-231 cells and examined the effects of LincK on EMT. The results showed that knockdown of LincK in MDA-MB-231 cells increased the expression of epithelial markers and decreased the expression of mesenchymal marker (Fig. 2j, k). Collectively, these data demonstrated that LincK promoted an EMT progress in breast cancer cells (BCC).

### LincK promoted migration and invasion in breast cancer cells

**Fig. 3:**
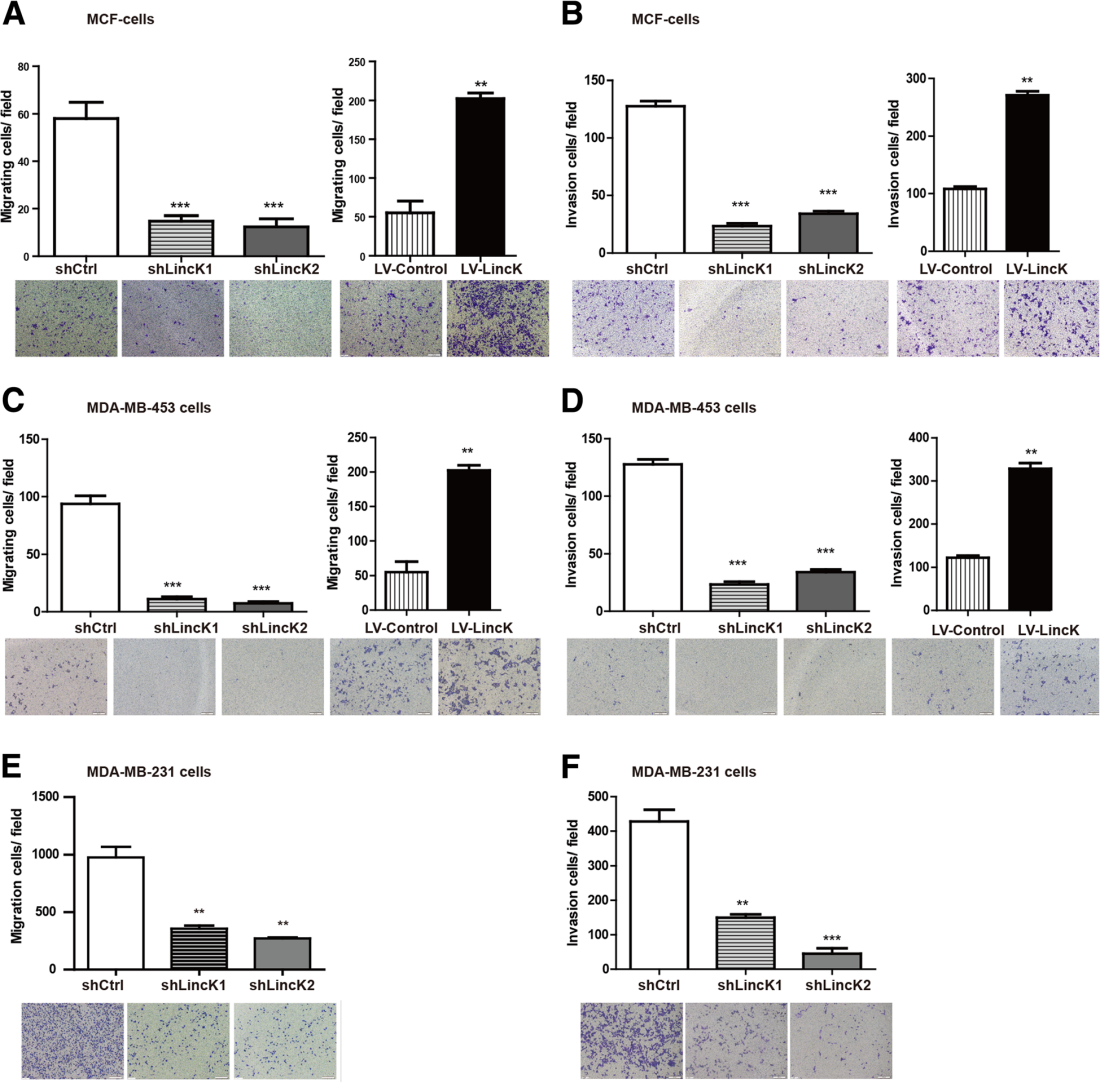
LincK promoted migration and invasion of breast cancer cells in vitro. **a**, **c**, **e** The effect of LincK on migration ability was measured using transwell assays in MCF-7 (**a**), MDA-MB-453 (**c**), and MDA-MB-231 (**e**). **b**, **d**, **f** The effect of LincK on invasion was measured using transwell assays in MCF-7 (**b**), MDA-MB-453 (**d**), and MDA-MB-231 (**f**). The data represent the mean number of cells per field and were presented as the means ± S.D. (***p < 0.001; **p < 0.01; *p < 0.05)). All the experiments were repeated three times

Next, we examined the changes in migration and invasion abilities after manipulating the expression of LincK. As weakly metastatic cell lines, MCF-7 and MDA-MB-453 are inert in migration and invasion under common culture condition [29, 30]. Thus, we chose to detect the migration and invasion abilities after co-culturing them with MSCs for 2 weeks. As shown in Fig. 3a–d, upregulating of LincK in MCF-7 cells and MDA-MB-453 increased migration and invasion, whereas suppressing LincK expression reduced migration and invasion compared with the corresponding control.

In invasive cell line MDA-MB-231, migration and invasion abilities were suppressed by knockdown of LincK (Fig. 3e, f). Collectively, these results indicated that LincK was able to significantly promote the migration and invasion of breast cancer cells.

### LincK enhanced proliferation and colony formation in BCC

**Fig. 4:**
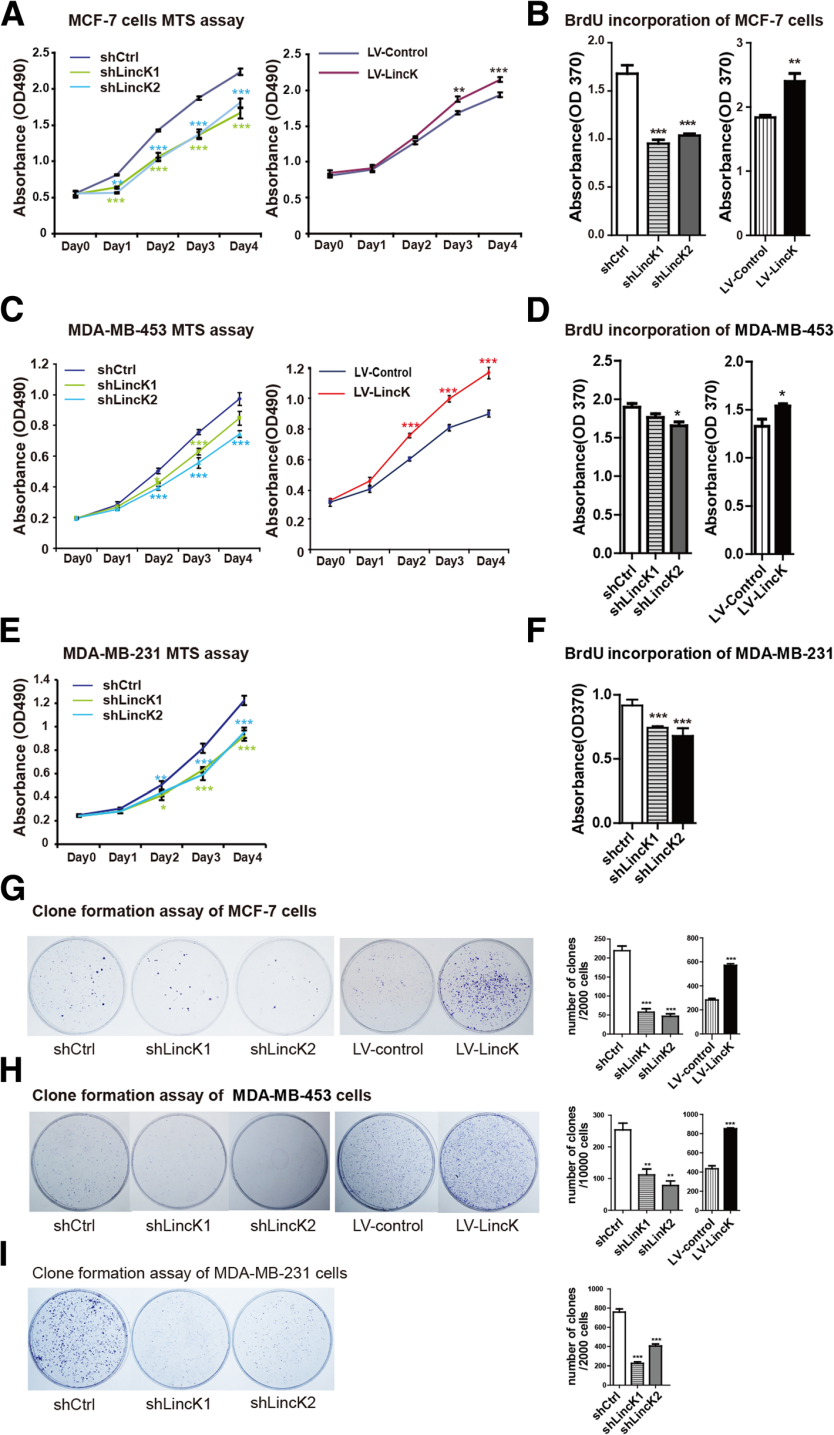
LincK promoted proliferation of breast cancer cells in vitro. **a**, **c**, **e** MTS assay in MCF-7 (**a**), MDA-MB-453 (**c**), and MDA-MB-231 (**e**) cells with LincK knockdown or overexpression. Representative results of triplicate experiments. Data were shown as means ± S.D. (*p < 0.05; **p < 0.01 by Student’s *t* test). **b**, **d**, **f** BrdU incorporation assay in MCF-7 (**b**), MDA-MB-453 (**d**), and MDA-MB-231 (**f**) with LincK knockdown or overexpression. Data were shown as means ± S.D. (*n* = 3, *p < 0.05; **p < 0.01 by Student’s *t* test). **g**, **h**, **i** Clone formation assay in MCF-7 (**g**), MDA-MB-453 (**h**), and MDA-MB-231 (**i**) with LincK knockdown or overexpression. Data showed mean ± S.D. of three independent experiments. (*p < 0.05; **p < 0.01 by Student’s *t* test)

To observe the effects of LincK on proliferation, we performed MTS assay and BrdU incorporation assay in BCC. MTS assay showed that knockdown of LincK significantly impaired cell growth and that overexpression of LincK considerably promoted cell growth (Fig. 4a, c, e). Further, we observed reduced BrdU incorporation in LincK-depleted cells and elevated BrdU incorporation in LincK-overexpressed cells (Fig. 4b, d, f).

Colony formation assay revealed that the clonogenic survival was greatly decreased following the knockdown of LincK, while markedly increased upon overexpression of LincK in BCC (Fig. 4g–i). These results suggested that LincK played an important role in regulating cell proliferation and colony formation.

### LincK positively regulated the expression of PBK and ZEB1

**Fig. 5:**
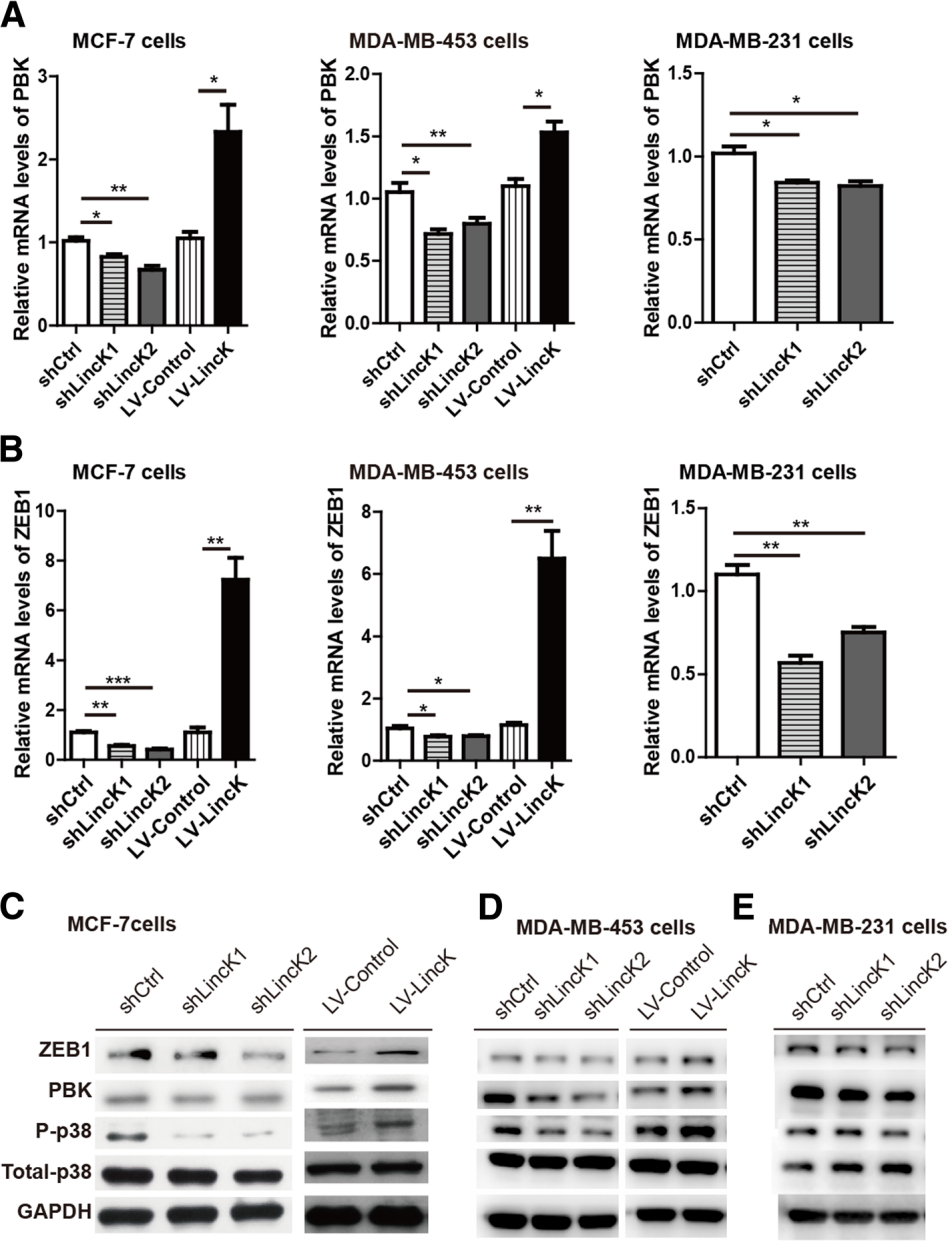
LincK positively regulated the expression of PBK and ZEB1. **a** qRT-PCR analysis of PBK expression level after LincK knockdown or overexpression in MCF-7 cells (left), MDA-MB-453 (middle), and MDA-MB-231 cells (right). **b** qRT-PCR analysis of ZEB1 expression level after LincK knockdown or overexpression in MCF-7 cells (left), MDA-MB-453 (middle), and MDA-MB-231 cells (right). **c**–**e** Western blot analysis of PBK, ZEB1, phospho-p38 MAPK, and p38 MAPK after LincK knockdown or overexpression in MCF-7 cells (**c**), MDA-MB-453 cells (**d**), and MDA-MB-231 cells (**e**). Data were shown as means ± S.D. *p < 0.05 and **p < 0.01 by Student’s *t* test

To detect the targeting of LincK, we used microarray to analyze changes in gene expression patterns after siRNA transfection in MCF-7 cells (Additional file 5: Figure S4A). Kyoto Encyclopedia of Genes and Genomes (KEGG) pathway analysis showed that the MAPK signaling pathway was differentially enriched after LincK-siRNAs transfection (Additional file 5: Figure S4B). Among the altered genes, we noticed that the expression of PBK significantly decreased after LincK knockdown. PBK, a serine-threonine kinase, was previously reported to phosphorylate p38 MAPK and promote tumor cell proliferation [31–33]. We therefore speculated that LincK mediated proliferation via PBK. As shown in Fig. 5a, knockdown of LincK resulted in the downregulation of the PBK transcript in the MCF-7, MDA-MB-453, and MDA-MB-231 cells. Western blot analysis demonstrated that the protein levels of PBK and its downstream target, phosphorylation of p38 MAPK, were decreased (Fig. 5c–e). Conversely, ectopic expression of LincK resulted in the upregulation of the PBK mRNA transcript as well as its protein levels and functional effects.

Because LincK was observed to induce EMT in BCC and ZEB1 has been proved to be a key regulator of EMT [10, 34], we aimed to determine whether LincK induced EMT by acting on the transcriptional factor ZEB1. Although we did not detect any statistically significant differences in ZEB1 immediately after siRNA transfection, we observed that the expression of ZEB1 decreased after stable shRNA-derived knockdown of LincK (Fig. 5b). Moreover, stable overexpression of LincK via lentivirus resulted in the upregulation of ZEB1 in MCF-7, MDA-MB-453 cells (Fig. 5c–e). Collectively, these data indicated that LincK could positively regulate the expression of PBK and ZEB1.

### LincK was physically associated with the miR-200 family

**Fig. 6:**
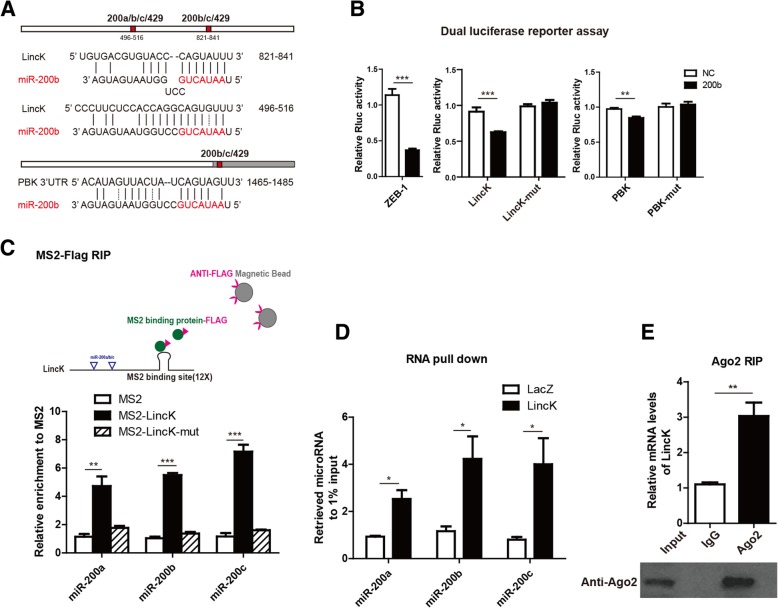
LincK was physically associated with miR-200 s. **a** Schematic outlining the predicted binding sites of miR-200 s on LincK. **b** Dual luciferase reporter assay in 293 T cells co-transfected with microRNAs (NC or miR-200b mimics) and psiCHECK2 (containing wild-type or mutant transcripts). Data were presented as the relative ratio of Renilla luciferase activity to Firefly luciferase activity. **c** Schematic outline of MS2-Flag RIP assay. 293 T cells were co-transfected by MS2bp-FLAG and MS2bs-LincK or MS2bs. Cell lysates were immunoprecipitated by FLAG M2 beads, and miR-200b endogenously associated with MS2bs-LincK were measured by qRT-PCR. Data were shown as means ± S.D. (*n* = 3). **d** RNA pull down assay of the binding of LincK and mir-200b. MCF-7 cell lysates were incubated with biotin-labeled LincK or LacZ, and the mir-200b expression level was detected by qRT-PCR after pull down. RNAs associated with biotin-LincK were compared to that of biotin-LacZ, and U6 was used as non-specific control. Data were shown as means ± S.D. (*n* = 3). **e** RIP assay of AGO2 enriched LincK in MCF-7 cells. IgG was used as negative controls. All relative abundances were compared to 1% input. Data were shown as means ± S.D. (*n* = 3). *p < 0.05; **p < 0.01; and ***p < 0.001 by two-tailed Student’s *t* test

Recently, several RNA transcripts have been reported to function as competing endogenous RNAs (ceRNA) by competitively binding microRNAs. To explore the underlying mechanism responsible for LincK in proliferation and EMT, we evaluated whether miRNAs were involved in the process. Bioinformatics analysis showed that LincK contained two putative binding sites for miR-200 s (Fig. 6a), which have been well-known regulators in inhibiting breast cancer proliferation, EMT, and metastasis [34, 35].

To validate whether LincK and PBK were bona fide targets of miR-200 s, such as ZEB1, we performed a dual luciferase reporter assay by inserting full-length or 3′UTR fragments containing wild-type or mutant miR-200 binding sites into the psiCHECK-2 vector. As a well-known miR-200 target, relative luciferase activity (RLA) of the ZEB1 construct can be significantly impaired when co-transfected with miR-200b. In line with ZEB1, the RLA of wild-type LincK and PBK 3′-UTR were markedly suppressed by miR-200b overexpression, while the reporter constructs containing mutant binding sites were unaffected (Fig. 6b).

To validate the direct binding between LincK and miR-200 s, we performed a RNA immunoprecipitation (RIP) analysis with MS2-binding protein-FLAG system (MS2bp), which specifically binds RNA containing MS2-binding sequences (MS2bs). The results showed that the mir-200b was enriched in LincK-MS2bs RNA in contrast to the negative control-MS2bs RNA (Fig. 6c).

To further validate the binding between miR-200 s and LincK at endogenous levels, we used in vitro transcribed biotin-labeled LincK to pull down endogenous microRNAs in the MCF-7 cells and examined using qRT-PCR. Compared with the control lacZ, LincK was observed to significantly enrich miR-200 s (Fig. 6d).

The microRNAs are known to bind their targets and cause translational repression and/or RNA degradation in an AGO2-dependent manner. To determine whether LincK was regulated by miR-200 s in such a manner, we conducted an anti-AGO2 RIP in the MCF-7 cells and found that endogenous LincK was specifically immunoprecipitated by Ago2 (Fig. 6e). Taken together, these data demonstrated that LincK was physically associated with miR-200 and may function as a ceRNA to affect miR-200 targets.

### LincK promoted tumorigenesis, growth, and metastasis of MCF-7 cells in nude mice

**Fig. 7:**
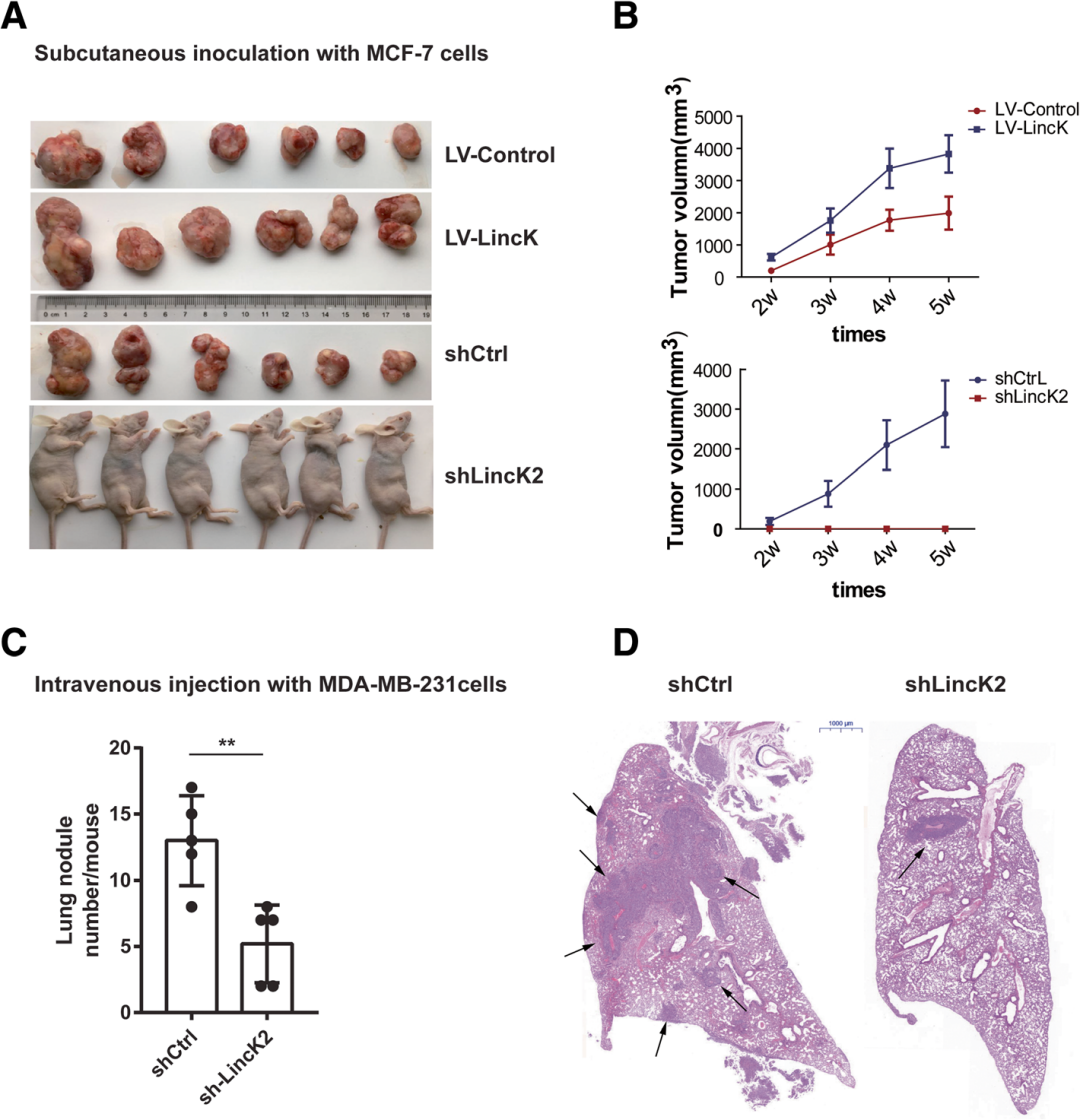
LincK promoted tumorigenesis, tumor growth, and metastasis in nude mice. **a**, **b** MCF-7 cells with LincK knockdown, overexpression, and the corresponding controls (stably transduced with lentivirus vectors) were mixed with hAD-MSCs at the ratio of 5:1 and inoculated subcutaneously into female nude mice. Tumorigenesis (**a**) and tumor growth (**b**) were examined by subcutaneous xenograft assay in nude mice. Tumor sizes were measured every week for 5 weeks; volumes (in cubic millimeters) were calculated according to the equation: width^2^ × length ×  0.5. *p < 0.05, Student’s *t* test. **c**, **d** MDA-MB-231 cells with LincK knockdown or control were injected intravenously into immunodeficiency mice. **c** Lung nodules at week 7 were analyzed as the number of nodules per mouse. **p < 0.01, Student’s *t* test. **d** Representative HE staining of lung sections was shown. Black arrowheads denoted representative nodules. Scale, 1000 μm

To further investigate the pathological relevance of LincK in vivo, we manipulated the expression of LincK in the MCF-7 cells and evaluated tumor growth and metastasis by using a xenograft tumor model. In our preliminary experiment, we subcutaneously injected five million MCF-7 cells into nude mice, and no tumors were formed after 10 weeks (*n* = 7). Next, we co-injected 1 × 10^6^ adipose derived-MSCs with 5 × 10^6^ MCF-7 cells into nude mice, and tumors were formed in most mice after 2 weeks (data not shown). Thus, we adopted the method of co-injecting of MSCs and MCF-7s into nude mice subcutaneously and measured the tumor volumes every week until sacrifice. Two weeks after tumor inoculation, palpable tumors were detected in all mice in the control group (shCtrl, *n* = 6). In sharp contrast, no tumors were formed in the LincK knockdown group (0/6, Fig. 7a). Overexpression of LincK formed clearly larger tumors than empty vectors did (Fig. 7b). As MCF-7 is non-metastatic cancer cell line, we did not detect metastatic foci in lung tissue sections by pathological examination of hematoxylin and eosin (HE) staining in all mice (data not shown).

To further evaluate the effects of LincK on metastasis, MDA-MB-231 cells (shCtrl or shLincK2) were transplanted into immunodeficient mice by tail vein injection. The rates of lung metastasis were comparable (6/6) in the two group observed at 7th week post-injection. The number of metastatic nodules in lungs was much less in mice injected with LincK ablation cells comparing with those of shCtrl mice (Fig. 7c, d). Collectively, these data indicated that LincK played a pivotal role in breast cancer tumorigenesis, growth, and metastasis.

### LincK was upregulated in breast cancer tissues

**Fig. 8:**
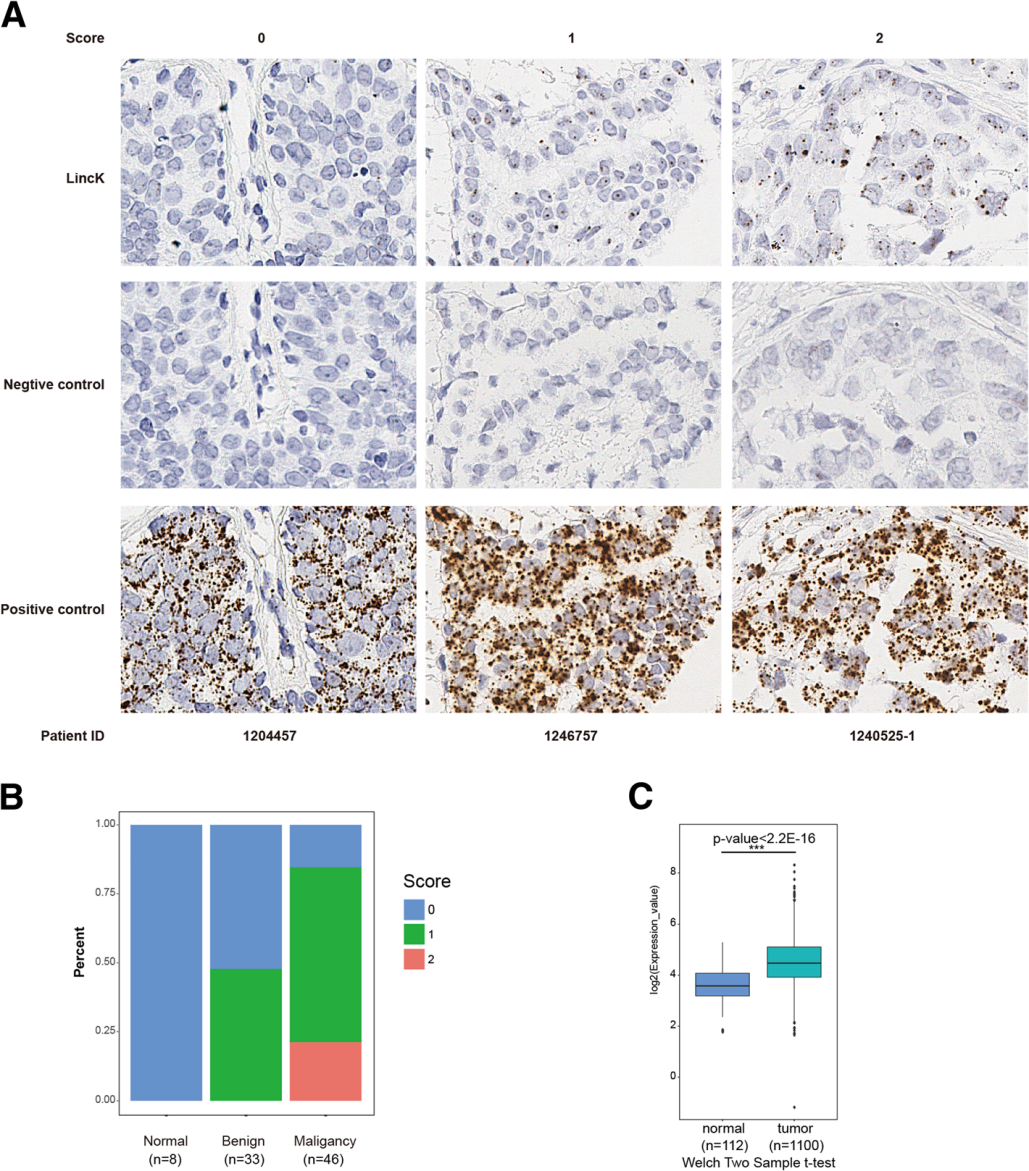
Expression of LincK in breast tissues and TCGA dataset. **a** Representative pictures of LincK scores detected by RNA Scope® technology. Breast tissues specimens consisting of normal, benign and invasive breast cancers (*n* = 87) were hybridized with probes against LincK (up), negative control (medium), and positive control (bottom). The brown dots indicated the target molecules (× 400 magnification). **b** Percentage of LincK scores (0, 1, and 2) in normal, benign, and malignancy breast tissues. **c** LincK expression was higher in breast cancer tissues compared with normal breast tissues from TCGA dataset. Significant differences were analyzed using the Welch two sample t-test (tumor, *n* = 1100 VS normal *n* = 112, *p* < 2.2e-16)

To explore if LincK was associated with clinical features, we constructed a tissue microarray (TMA) composed of 87 specimens (8 healthy mammary, 33 hyperplasia/fibroma, and 46 invasive breast cancer). The transcriptional levels of the endogenous LincK gene were examined by in situ hybridization (RNAscope®). LincK was barely expressed in healthy mammary tissues and was expressed at a relatively modest level in benign tissues. Upregulation of LincK was more frequently observed in the breast cancer patients (Fig. 8b, Additional file 1: Table S2, *P* = 0.000). With the limited sample size (*n* = 46), statistical analyses showed no significant correlation between the expression of LincK and tumor size or advanced clinical staging (Additional file 1: Table S3).

To further verify the correlation of LincK with breast cancer, we analyzed RNA-seq data in The Cancer Genome Atlas (TCGA) by bioinformatic methods. The results revealed that LincK was significantly upregulated in breast cancer tissues than in normal breast tissues (*p* < 2.2e-16, Fig. 7c), which was consistent with our observation.

## Discussions

In the current study, we screened for functional lncRNAs in the MSCs-induced EMT by using microarray assay and identified a functional lncRNA (LincK). LincK can be upregulated by co-culture-induced EMT and by other EMT activators such as IL-6 and TGF-β. LincK promotes proliferation and EMT in breast cancer cells in vitro as well as tumor growth in immunodeficient mice. More importantly, LincK was frequently elevated in breast cancer compared with normal breast tissue in clinical samples.

It has been shown that some lncRNAs, dominantly localized in the cytoplasm, can act as a ‘sponge’ via the competitive binding of common miRNA and attenuating the suppression of miRNAs on protein-coding RNAs [36, 37]. In this study, we reported that LincK functions in a similar way. LincK contained two putative miR-200 binding sites and was observed to compete with their endogenous targets ZEB1 and PBK. By modulating the level of LincK, we observed that the expression of ZEB1 and PBK highly correlated with the expression of LincK. The miR-200 family has been found to be deregulated in multiple types of cancers and play a pivotal role in tumor initiation, maintenance and malignant metastasis [35, 38, 39].

LincK contains an Alu element in 3′ end. Recent studies have shown that some Alu element-containing lncRNAs, which were largely cytoplasmic and polyadenylated, might be involved in Staufen1 (STAU1)-mediated mRNA decay (SMD) [40, 41]. During SMD, lncRNA could regulate the degradation of the mRNA target by forming dsRNA structures between the imperfect pairing of their Alu elements. We could not exclude the possibility that the Alu-containing lncRNA LincK plays a crucial role in SMD.

In this study, we used three breast cancer cell lines to investigate the function of LincK in vitro. For enhancing proliferation, LincK displayed a similar role in the three cell lines. However, for promoting EMT, LincK regulated the morphology of three cell lines differently. In MCF-7 cells, autonomous EMT was exhibited after ectopic expression of LincK by lentivirus infection and antibiotic selection for 2 weeks (Fig. 2d). In MDA-MB-453 cells, no obvious morphological changes were observed by simply regulating the expression of LincK. After co-culture with MSCs (Fig. 2g) or treatment with TGF-β (data not shown), the difference in EMT features between LincK overexpression and the controls became apparent. In mesenchymal-like MDA-MB-231 cells, depletion of LincK caused changes in expression of EMT markers while not in the shapes of cells. However, the migration and invasion abilities were clearly correlated with the expression of LincK in the three cell lines. These data demonstrated that LincK consistently promoted EMT progress in BCC, although it triggered morphological changes differently upon cell context.

Using the subcutaneous model, we showed that silencing of LincK dramatically blocked tumor growth and that the ectopic expression of LincK promoted tumor growth. This was consistent with the observation that LincK played a positive role in proliferation in vitro. Although these cells underwent EMT accompanied by increased migration and invasion capacity in vitro, we did not detect lung metastasis in subcutaneous model. There might have been at least three reasons responsible for the discrepancy. First, MCF-7 cells showed relatively low metastasis in vivo [42]. Second, the occurrence of metastasis in mouse models using human breast cancer cells was low especially when cells were implanted subcutaneously [43–45]. Another possible reason was that LincK was not sufficient enough to trigger metastasis in vivo. Using lung metastasis model by intravenous injection of MDA-MB-231 cells, we found knockdown of LincK significantly impaired the lung metastasis of MDA-MB-231 cells. Other models, such as orthotopic incubation or ventricle injection, are useful for further investigating the function of LincK on metastasis in vivo.

In this study, we used RNAscope® technology to detect the expression of LincK in clinical specimens. Despite limited cases and short-term observation, we proved that LincK was differently expressed among normal and breast cancer tissues. The results obtained from the open database TCGA additionally confirmed our findings. It should be noted that we did not observe a significant relation between LincK expression and metastasis in our clinical samples. All data suggested that it is important to conduct more detailed and long-term investigations on relationships between LincK expression and clinical features including histological subtype, treatment response, and survival.

Recently, Gasri-Plotnitsky et al. reported that lncRNA GASL1, transcribed from the same chromosome position as LincK, functions as tumor suppressor by inhibiting cell proliferation in osteosarcoma and lung carcinoma cells [17]. In this study, we proved that LincK was upregulated in breast cancer patients and promoted growth and metastasis in breast cancer cells. GASL1 is 1536 base long consisting of four exons, and LincK is 1210 base long consisting of two exons (Fig. 1f). Although partly overlap between GASL1 and LincK, they confer different even opposite effects depending on different tissues or diseases.

Recently, increasing studies have demonstrated that MSCs within the tumor stroma may promote breast cancer progression by secreting cytokines such as CCL5, IL-6, or TGF-β [7–9]. In addition to paracrine cytokines, MSCs can trigger a select set of miRNAs in BCC and promote breast cancer metastasis. However, currently, the lncRNAs underlying how MSCs contribute to tumor pathogenesis remain incompletely understood. Our data demonstrate that LincK plays a role in tumor-hADMSC interaction. Co-culture of tumors and hAD-MSCs provide an easy and useful platform for mimicking tumor microenvironments and can identify important members involved in tumor progress. Given the specific expression pattern and significant role of LincK in tumor growth and metastasis, we propose that LincK is a promising diagnostic biomarker or therapeutic target for human breast cancer. Recently, great advancements have been made in design and modification of lncRNAs inhibitors such as siRNAs, antisense oligonucleotides, and small molecular, which laid the foundation for lncRNA-based cancer therapy. However, there are still some obstacles that need to be overcome before their clinical application, including specific delivery to the target tissue or cell, stability, and toxicity control.

## Conclusion

LncRNAs are promising candidates for new diagnostic biomarkers and therapeutic targets due to their enormous quantity throughout the genome, specific expression patterns, as well as versatile functions and flexible mechanisms. We reported here that the lncRNA LincK plays a significant role in regulating of EMT and tumor growth and that it could be a potential therapeutic target in breast cancers.

## Additional files

Additional file 1: **Table S1.** Nucleotide sequence of primers, probes and siRNAs used in this study. Table S2 Correlation of LincK expression in breast tissue samples (*n* = 87). Table S3 Correlation of LincK expression in breast cancer tissue with clinicopathological characteristics. (DOCX 28 kb)

Additional file 2: **Figure S1.** Bioinformatics analysis of differential expressed coding genes (A) KEGG pathway analysis of differential expressed coding gene in MCF7 cells co-cultured with hAD-MSCs versus cultured alone. (B) Heat map representation of microarray data about the expression levels of EMT-related genes in MCF7 cells co-cultured with hAD-MSCs versus cultured alone. (C) GSEA analysis of differential expressed mRNAs in previous published datasets. These mRNAs were enriched in TGF-β associated gene sets. The description of the gene sets and the nominal *p* values were shown. (D) Pie chart of differential expressed lncRNA types in MCF7 cells co-cultured with hAD-MSCs versus cultured alone. (E) The nucleotide sequence of full-length human LincK. (TIF 1144 kb)

Additional file 3: **Figure S2.** Bioinformatics analysis of coding potential of LincK (A) The coding potential of LincK predicted by three published software. NEAT1 used as non-coding RNA control and GAPDH used as coding RNA control. (B) Ratio of reads per kilobase million (RPKM) values from ribosomal profiling and input-RNA profiling of each indicated gene in Hela. NEAT1 and MALAT1 were used as noncoding RNA control. GAPDH and ACTB were used as coding RNA control. (TIF 142 kb)

Additional file 4: **Figure S3.** Knockdown of LincK inhibited EMT programs in MCF-7 cells induced by co-cultured with hAD-MSCs. (A) qRT-PCR assay of EMT markers in MCF-7 (shCtrl or shLincK2) after co-culture with hAD-MSCs for two weeks. Data were shown as means ± S.D. (*n* = 3). Statistical differences were analyzed using Student’s test (**p < 0.01). (B) Western Bolt assay of EMT markers in MCF-7 cells (shCtrl or shLincK2) after co-culture with hAD-MSCs for two weeks. (TIF 177 kb)

Additional file 5: **Figure S4.** Microarray-based expression-profile analysis in MCF7 cells transfected with si-LincK1 and si-LincK2 and compared with si-negative control (NC). (A) The heatmap represented the differential expressed genes after siRNAs mediated LincK knockdown. All three replicates were shown. Black arrowhead denotes PBK. (B) KEGG analysis of differentially expressed genes in MCF-7 cells transfected with si-LincKs versus si- NC, categorized by molecular function (MF). (TIF 309 kb)

## Abbreviations

BCC: Breast cancer cells
ELISA: Enzyme-linked immunosorbent assay
EMT: Epithelial-mesenchymal transition
GSEA: Gene set enrichment analysis
KEGG: Kyoto Encyclopedia of Genes and Genomes
lncRNAs: Long noncoding RNAs
MSC: Mesenchymal stem cells
qRT-PCR: Quantitative reverse transcription-polymerase chain reaction
RACE: Rapid amplification of cDNA ends
