# Supplementary material for: LincK contributes to breast tumorigenesis by promoting proliferation and epithelial-to-mesenchymal transition
Source: J Hematol Oncol. 2019 Feb 22;12:19. doi: 10.1186/s13045-019-0707-8 (PMC6387548; doi:10.1186/s13045-019-0707-8)
Supplement: Supplementary file 1 — Table S1. Nucleotide sequence of primers, probes and siRNAs used in this study. Table S2 Correlation of LincK expression in breast tissue samples (n = 87). Table S3 Correlation of LincK expression in breast cancer tissue with clinicopathological characteristics. (DOCX 28 kb) [file 13045_2019_707_MOESM1_ESM.docx]

Table S1. Nucleotide sequence of primers, Probes and siRNAs used in this study.

| Primer names | Sequences |
| --- | --- |
| Vim-qF | TGCCGTTGAAGCTGCTAACTA |
| Vim-qR | CCAGAGGGAGTGAATCCAGATTA |
| CDH1-qF | TGCCCAGAAAATGAAAAAGG |
| CDH1-qR | GTGTATGTGGCAATGCGTTC |
| CDH2-qF | ACAGTGGCCACCTACAAAGG |
| CDH2-qR | CCGAGATGGGGTTGATAATG |
| ZO1-qF | ACCAGTAAGTCGTCCTGATCC |
| ZO1-qR | TCGGCCAAATCTTCTCACTCC |
| hGAPDH-qF | TGCCATCAATGACCCCTTC |
| hGAPDH-qR | CATCGCCCCACTTGATTTTG |
| U1-qF | GGGAGATACCATGATCACGAAGGT |
| U1-qR | CCACAAATTATGCAGTCGAGTTTCCC |
| ZEB1-qF | CAGCTTGATACCTGTGAATGGG |
| ZEB1-qR | TATCTGTGGTCGTGTGGGACT |
| PBK-qF | TAGGAGTCTCTCTACCACTGGA |
| PBK-qR | TCCCACAAAGTAAGGCCAAAG |
| ZEB1-3UTR-F | GCCTGAACCTCAGACCTAGTA |
| ZEB1-3UTR-R | GCTTAAGGCCAAAGGAGATACA |
| PBK-3UTR-F | GAAGTGTGGCTTGCGTAAATAA |
| PBK-3UTR-R | CTGGTACCCAAAGTGTCCTTTA |
| LincK-qF | CTACAGGGTTCATAGAGGAGGA |
| LincK-qR | CAGAGCTTCCTGAGTCCTTTAG |
| Mutant-PBK | 3’UTR with ‘ATAGTTACTATCAGTAGT’ deletion |
| Mutant-LinK | Full length with ‘ACCAGGCAGTGTT’ and ‘GTACCCAGTATT’ deletion |
| Full length-LincK- F | AGACCGCCACAGAAGAGT |
| Full length-LincK- R | ACGGCTAAATAGTACTCCATTAGTATAG |
| 5’RACE Outer-F | TTAGCGCTGAAAGTCAGAGAG |
| 5’RACE Inner-F | CCTCCTCTATGAACCCTGTAGA |
| 3’RACE Outer-R | AAGTTTCCAACGGGAAGGG |
| 3’RACE Inner-R | CTGAGGCTGAGAGACATGTAATAA |
| siRNA-Link1 | r(GCUGCAAGGGAAAUGACAU)dTdT |
| siRNA-Link2 | r(GGCUGAGAGACAUGUAAUA)dTdT |
| LNA probe For LincK detected by North Blot and FISH | /5DigN/ACATGCTTTCTCTCTGGGTTTA/3Dig_N/ |
| RT-mir200a-3p | GTCGTATCCAGTGCAGGGTCCGAGGTATTCGCACTGGATACGACACATCGT |
| SENSE-mir200a-3p | CTGGAGTAACACTGTCTGGTAA |
| RT-mir200b-3p | GTCGTATCCAGTGCAGGGTCCGAGGTATTCGCACTGGATACGACTCATCAT |
| SENSE-mir200b-3p | CTGGAGTAATACTGCCTGGTAA |
| RT-mir200c-3p | GTCGTATCCAGTGCAGGGTCCGAGGTATTCGCACTGGATACGACTCCATCA |
| SENSE-mir200c-3p | CTGGAGTAATACTGCCGGGTAA |
| Consensus-Antisense for miRNAs | GTGCAGGGTCCGAGGT |
| U6 RT primer | AAAATATGGAACGCTTCACGAATTTG |
| U6-qF | CTCGCTTCGGCAGCACATATACT |
| U6-qR | ACGCTTCACGAATTTGCGTGTC |

Table S2 Correlation of LincK expression in breast tissue samples (n=87)

| Characteristics | LincK | | | P value |
| --- | --- | --- | --- | --- |
|  | 0 | 1 | 2 |  |
| Normal | 8 | 0 | 0 | 0.000 |
| Benign | 17 | 16 | 0 |  |
| Maligancy | 7 | 29 | 10 |  |

Table S3 Correlation of LincK expression in breast cancer tissue with clinicopathological characteristics

| Maligancy | LincK | | | P value |
| --- | --- | --- | --- | --- |
| (n=46) | 0 | 1 | 2 |  |
| Tumor size |  |  |  |  |
| <3cm | 3 | 20 | 5 | 0.325 |
| >3cm | 4 | 9 | 5 |  |
| Stage |  |  |  |  |
| I/Tis | 2 | 12 | 3 |  |
| II | 4 | 17 | 5 | 0.211 |
| III/IV | 1 | 0 | 2 |  |
| Histology |  |  |  |  |
| Well | 0 | 4 | 1 | 0.607 |
| Moderately | 4 | 17 | 4 |  |
| Poorly | 3 | 8 | 5 |  |
| Ki67 |  |  |  |  |
| >50% | 2 | 8 | 5 | 0.415 |
| <50% | 5 | 21 | 5 |  |
| Age |  |  |  |  |
| >50 | 3 | 16 | 4 | 0.653 |
| <50 | 4 | 13 | 6 |  |
| ER |  |  |  |  |
| positive | 5 | 16 | 4 | 0.436 |
| negtive | 2 | 13 | 6 |  |
| PR |  |  |  |  |
| positive | 3 | 14 | 3 | 0.603 |
| negtive | 4 | 15 | 7 |  |
| Her2 |  |  |  |  |
| 0/+ | 5 | 11 | 4 | 0.267 |
| ++/+++ | 2 | 18 | 6 |  |

Fisher exact test
