# Supplementary figures and images for: LincK contributes to breast tumorigenesis by promoting proliferation and epithelial-to-mesenchymal transition
Source: J Hematol Oncol. 2019 Feb 22;12:19. doi: 10.1186/s13045-019-0707-8 (PMC6387548; doi:10.1186/s13045-019-0707-8)

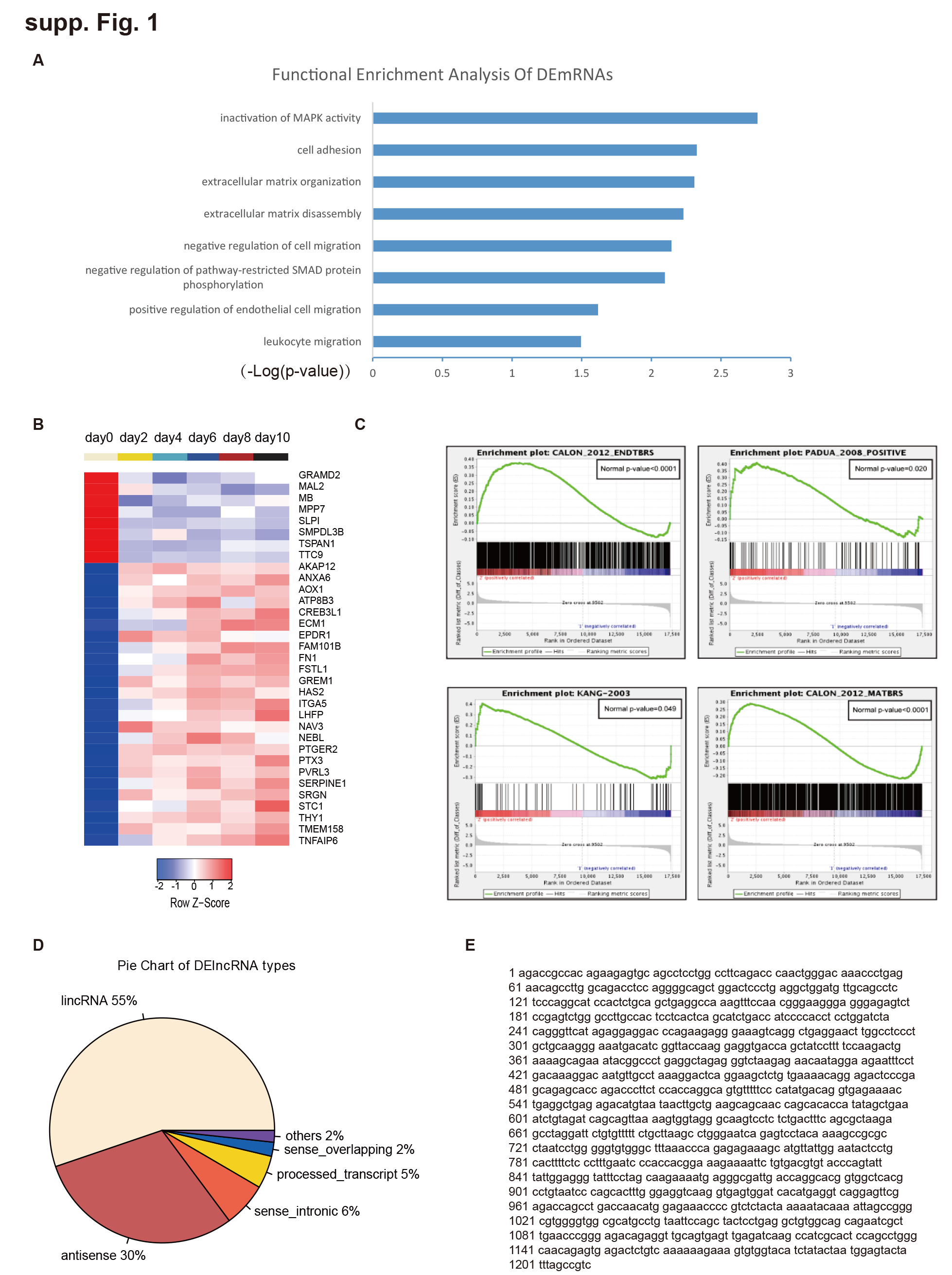

Supplement: Supplementary file 2 — Figure S1. Bioinformatics analysis of differential expressed coding genes (A) KEGG pathway analysis of differential expressed coding gene in MCF7 cells co-cultured with hAD-MSCs versus cultured alone. (B) Heat map representation of microarray data about the expression levels of EMT-related genes in MCF7 cells co-cultured with hAD-MSCs versus cultured alone. (C) GSEA analysis of differential expressed mRNAs in previous published datasets. These mRNAs were enriched in TGF-β associated gene sets. The description of the gene sets and the nominal p values were shown. (D) Pie chart of differential expressed lncRNA types in MCF7 cells co-cultured with hAD-MSCs versus cultured alone. (E) The nucleotide sequence of full-length human LincK. (TIF 1144 kb) [file 13045_2019_707_MOESM2_ESM.tif]

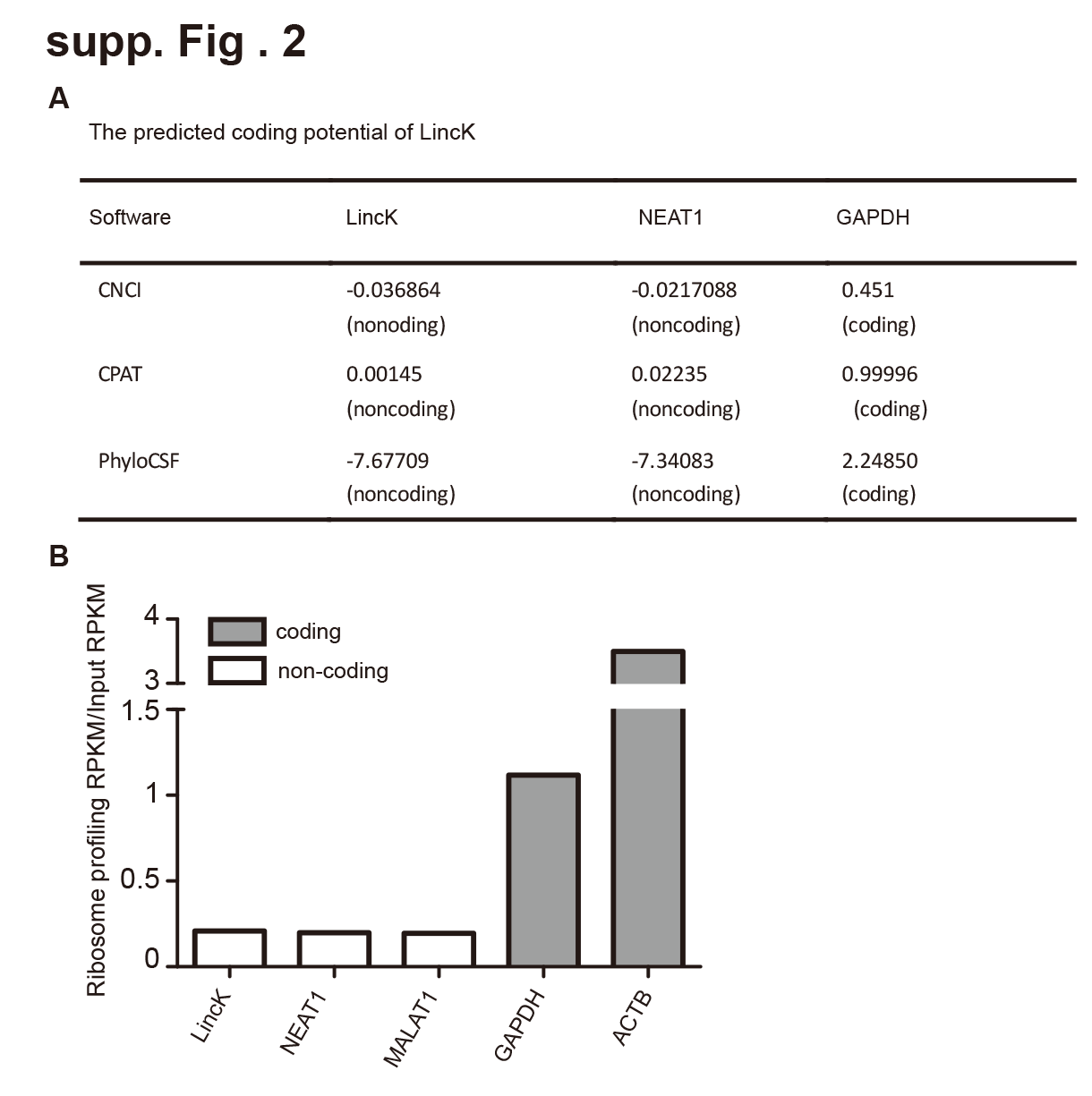

Supplement: Supplementary file 3 — Figure S2. Bioinformatics analysis of coding potential of LincK (A) The coding potential of LincK predicted by three published software. NEAT1 used as non-coding RNA control and GAPDH used as coding RNA control. (B) Ratio of reads per kilobase million (RPKM) values from ribosomal profiling and input-RNA profiling of each indicated gene in Hela. NEAT1 and MALAT1 were used as noncoding RNA control. GAPDH and ACTB were used as coding RNA control. (TIF 142 kb) [file 13045_2019_707_MOESM3_ESM.tif]

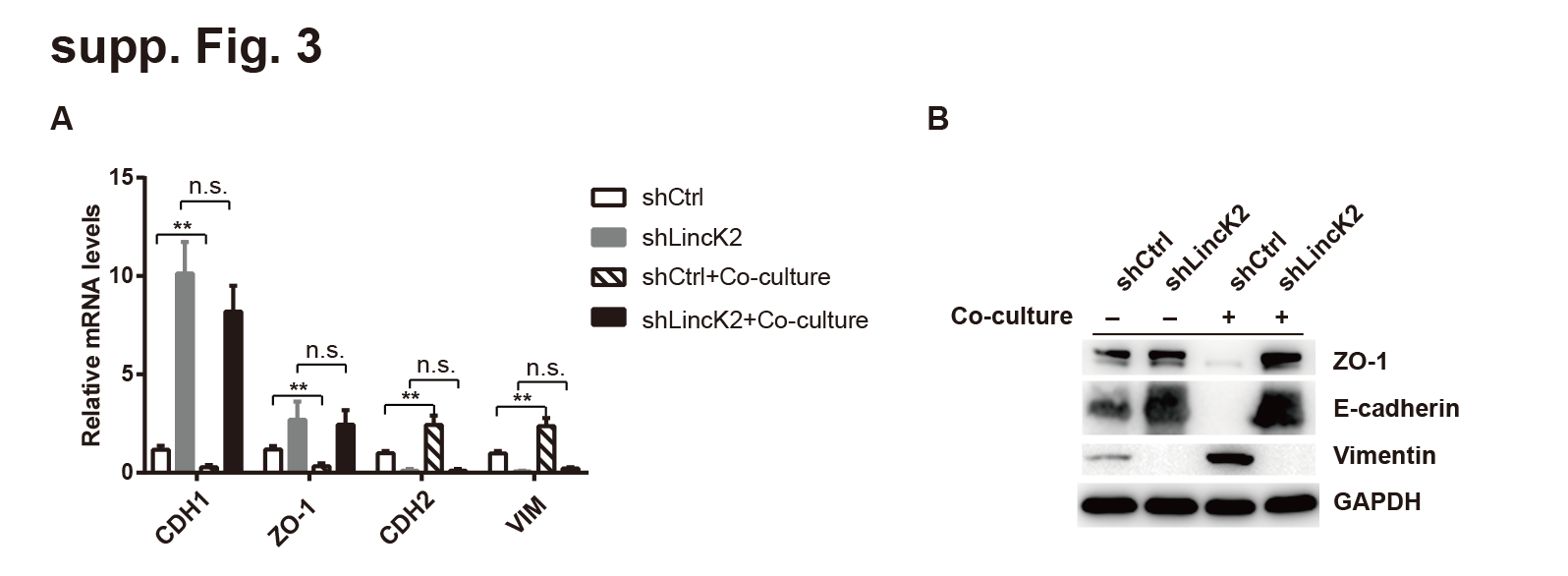

Supplement: Supplementary file 4 — Figure S3. Knockdown of LincK inhibited EMT programs in MCF-7 cells induced by co-cultured with hAD-MSCs. (A) qRT-PCR assay of EMT markers in MCF-7 (shCtrl or shLincK2) after co-culture with hAD-MSCs for two weeks. Data were shown as means ± S.D. (n = 3). Statistical differences were analyzed using Student’s test (**p < 0.01). (B) Western Bolt assay of EMT markers in MCF-7 cells (shCtrl or shLincK2) after co-culture with hAD-MSCs for two weeks. (TIF 177 kb) [file 13045_2019_707_MOESM4_ESM.tif]

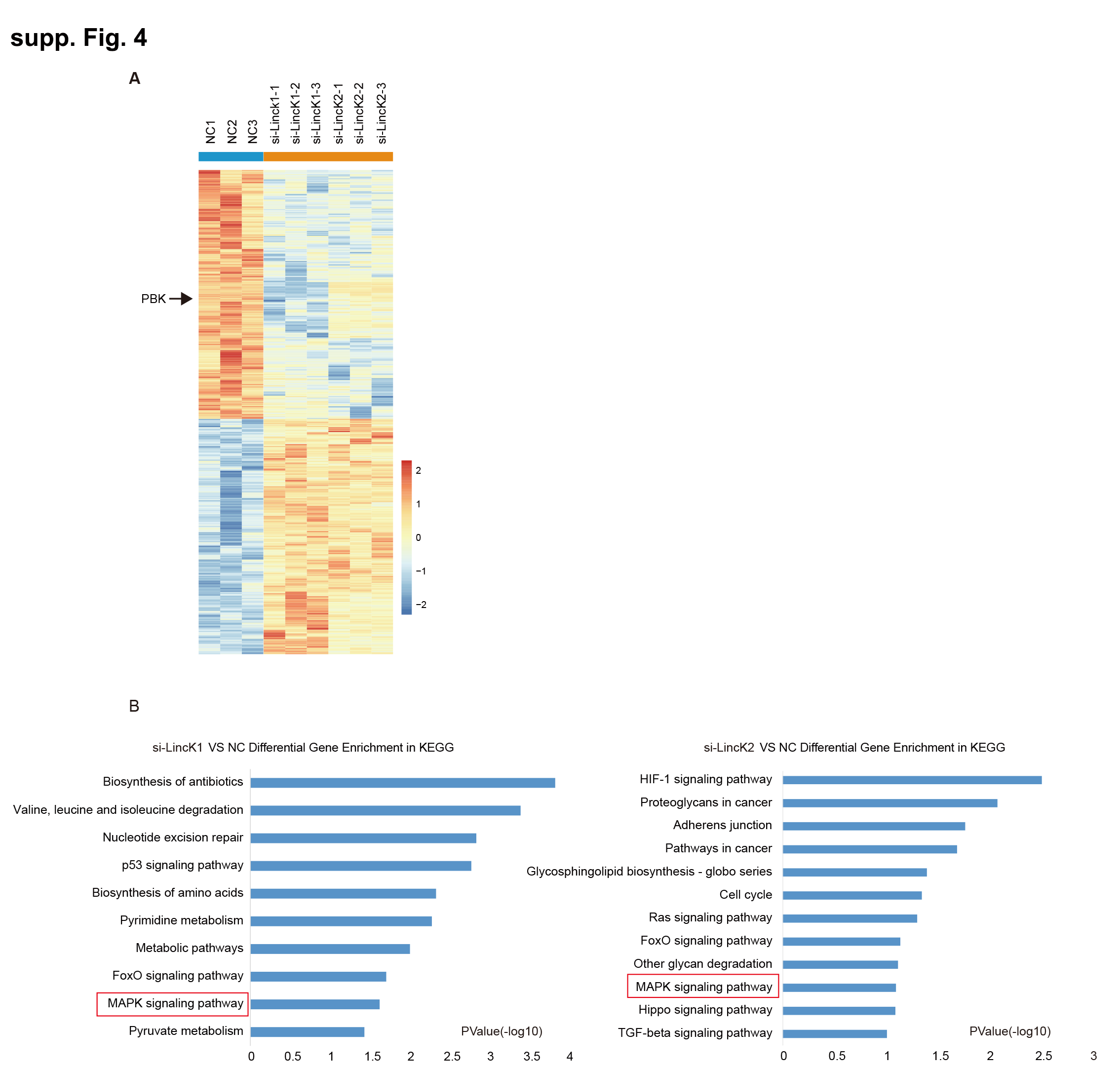

Supplement: Supplementary file 5 — Figure S4. Microarray-based expression-profile analysis in MCF7 cells transfected with si-LincK1 and si-LincK2 and compared with si-negative control (NC). (A) The heatmap represented the differential expressed genes after siRNAs mediated LincK knockdown. All three replicates were shown. Black arrowhead denotes PBK. (B) KEGG analysis of differentially expressed genes in MCF-7 cells transfected with si-LincKs versus si- NC, categorized by molecular function (MF). (TIF 309 kb) [file 13045_2019_707_MOESM5_ESM.tif]
